# Symptomatic and Incidental Venous Thromboembolic Disease Are Both Associated with Mortality in Patients with Prostate Cancer

**DOI:** 10.1371/journal.pone.0094048

**Published:** 2014-08-15

**Authors:** Shruti Chaturvedi, Surbhi Sidana, Paul Elson, Alok A. Khorana, Keith R. McCrae

**Affiliations:** 1 Department of Internal Medicine, Cleveland Clinic, Cleveland, Ohio, United States of America; 2 Department of Quantitative Health Sciences, Cleveland Clinic, Cleveland, Ohio, United States of America; 3 Department of Cellular and Molecular Medicine, Cleveland Clinic, Cleveland, Ohio, United States of America; 4 Taussig Cancer Institute, Cleveland Clinic, Cleveland, Ohio, United States of America; Maastricht University Medical Center, Netherlands

## Abstract

**Introduction:**

The association between malignancy and venous thromboembolic disease (VTE) is well established. The independent impact of VTE, both symptomatic and incidental, on survival in patients with prostate cancer is not known. We conducted a retrospective cohort study to evaluate the effect of VTE of survival in prostate cancer.

**Methods:**

Data regarding clinical characteristics, treatment and outcomes of 453 consecutive prostate cancer patients were collected. Fisher exact (categorical variables) and t-test (continuous variables) were utilized to test associations with VTE and mortality. Survival was estimated using the Kaplan Meier method. A Cox regression model was used to model the mortality hazard ratio (HR).

**Results:**

At diagnosis, 358 (83%) patients had early stage disease, 43 (10%) had locally advanced disease and 32 (7%) had metastatic disease. During the follow up period, 122 (27%) patients died and 41 (9%) developed VTE (33 deep vein thrombosis, 5 pulmonary embolism, and 3 patients with both DVT and PE). Twenty-five VTE events were symptomatic and 16 were incidentally diagnosed on CT scans obtained for other reasons. VTE was associated with increased mortality [HR 6.89 (4.29–11.08), p<0.001] in a multivariable analysis adjusted for cancer stage, performance status, treatments and co-morbidities. There was no difference in survival between patients who had symptomatic and incidental VTE.

**Conclusion:**

Venous thromboembolic disease, both symptomatic and incidental, is a predictor of poor survival in patients with prostate cancer, especially those with advanced disease. Further studies are needed to evaluate the benefit of prophylactic and therapeutic anticoagulation in this population.

## Introduction

The association between thrombosis and malignancy was first reported by Trousseau in 1865 [1]. Since then, many studies have confirmed the increased incidence of venous thromboembolism (VTE) in patients with cancer [2], [3] as well as the development of clinically overt cancer after an episode of apparently idiopathic VTE [4]. Several recent studies have reported that VTE is associated with higher mortality in patients with cancer due to direct effects of VTE as well as other causes, including progressive disease [5]–[8]. Moreover, preclinical data indicate that activation of the coagulation system is integrally linked with tumor progression through direct effects on angiogenesis and metastasis [9]–[12]. These observations suggest that the development of the clinical hypercoagulable state may be a surrogate of aggressive tumor biology and poor prognosis.

The association between thrombosis and cancer-related outcomes has been studied in several malignancies, for example pancreatic and other gastrointestinal cancers that are associated with high risk of VTE. Prostate cancer is typically associated with a lower risk of thrombosis [13] although it presents a significant burden of disease, with 238,000 new cases diagnosed annually in the United States [14]. The effect of VTE on survival in patients with prostate cancer is not known. Furthermore, it is not known whether traditional risk factors for thrombosis in patients without malignancy, for example obesity, smoking and CHF modify the risk of VTE in these patients.

The widespread use of multi-detector CT scans for staging in patients with cancer has led to an emerging problem of clinically asymptomatic, incidentally diagnosed pulmonary embolism and deep vein thrombosis [15],[16]. Recent reports suggest that the clinical characteristics of patients with incidental VTE are similar to those with symptomatic VTE, and that these events are associated with higher rates of recurrent VTE, hemorrhage and mortality [17], [18].

We conducted a retrospective cohort study to estimate the impact of VTE, both symptomatic and incidental, on survival in patients with prostate cancer. We further attempted to identify risk factors for thrombosis in patients with prostate cancer.

## Methods

We initially identified 465 consecutive patients who received clinical care for prostate cancer from January 1, 2006 to June 30, 2006 at the Cleveland Clinic. Patients who were seen for a single consultation and those for whom follow up data was not available were excluded from the analysis. The final study cohort comprised 453 men with prostate cancer. Electronic medical records, including all imaging studies were reviewed for demographic and clinical data including age, date of diagnosis (defined as date of first pathologic confirmation of prostate cancer, or date of first clinical encounter if unavailable), stage, Gleason grade, details of treatment (radiation, surgery, hormonal treatment and chemotherapy), date of VTE, and date of death, where applicable. VTE was identified on the basis of ICD-9 diagnosis codes, clinical documentation and review of imaging (venous Doppler and CT scans). For each episode of VTE, records were examined to identify the site, setting (outpatient versus hospitalized), relation to concurrent surgery, radiation, hormonal or chemotherapy (within 4 weeks), and the presence of symptoms consistent with VTE such as extremity swelling, tenderness or pain, chest pain, shortness of breath or cough. Patients were followed until death or loss to follow up. For surviving patients, data was censored at the date of the last clinical encounter.

Continuous variables were summarized by means and standard deviations and categorical variables by counts and proportions. Factors associated with VTE were evaluated by uni-variate followed by multivariate analysis. Factors associated with mortality were examined in an identical manner. Survival functions were estimated using the Kaplan Meier method and a Cox regression model was used to model the mortality hazard ratio with date of diagnosis as the time origin. This model adjusted for potential confounders including stage of disease, treatments received, ECOG performance status, age and other co-morbidities such as congestive heart failure, diabetes mellitus, renal disease, chronic obstructive pulmonary disease, peripheral vascular disease, and the presence of other cancers. To account for the timing of the VTE in the survival analyses it was treated as a time-varying covariate. Statistical analysis was performed using SPSS version 20 (IBM Corp, USA). All p – values and 95% confidence intervals were 2 sided and all tests were conducted at the 0.05 level of significance.

This Institutional Review Board at the Cleveland Clinic approved this study. Patient confidentiality was maintained by de-identifying patient data after collection and maintaining data in RedCap, a secure, electronic data management system hosted at Cleveland Clinic.

## Results

Our study cohort comprised 453 men with prostate cancer. Table 1 summarizes the clinical characteristics of these patients. Median age at diagnosis was 66 years (range 42–90 years). Three hundred and fifty eight (83%) patients had early stage disease, 43 (10%) had locally advanced disease and 32 (7%) had metastatic disease at diagnosis. The most frequently encountered comorbidities were another malignancy (n = 103), diabetes mellitus (n = 89) and atrial fibrillation. Of the 103 patients with another malignancy (33 lung cancer, 24 colorectal cancer, 12 pancreatic cancer, 15 bladder cancer, 12 renal cell cancer, 7 esophageal cancer, 12 lymphomas, 11 hepatocellular cancer, 2 neuroendocrine tumors, 2 glioblastoma multiforme, 1 acute myeloid leukemia and 3 adenocarcinomas of unknown primary) the alternate cancer was the primary cause of death in 35 (34%).

**Table 1:** Demographics and clinical characteristics of patient cohort.

	N (%)
Median age	66 years (42–90 years)
**Stage of disease**	
Localized disease	358 (83)
Locally advanced disease	43 (10)
Metastatic disease	32 (7)
**Treatment modalities received**	
Hormonal therapy	196 (43)
Chemotherapy	69 (15)
Radiotherapy	322 (71)
Surgery	98 (22)
**Comorbidities**	
Atrial Fibrillation	45 (10)
Congestive Heart Failure	39 (9)
Peripheral Vascular Disease	22 (5)
COPD	40 (9)
Dementia	13 (3)
Diabetes Mellitus	89 (20)
Chronic Kidney Disease	34 (8)
Rheumatologic Disease	15 (3)
Hip fracture	1 (<1)
Hypercoagulable disorder	3 (1)
Other Malignancy	103 (23)
Incidence of VTE	41 (9)
Mortality	122 (27)
Estimated median survival	14.7 years

Twenty two percent of patients were treated with a prostatectomy, 70% received radiation therapy, 43% were treated with hormonal therapy (most commonly LHRH agonists and anti-androgens, alone or in combination) and 15% patients received chemotherapy. Median follow up time was 83 months (25–75% 56–90 months). One hundred and twenty-two (27%) patients died during the period of follow up. Estimated median survival was 14.7 years from diagnosis.

### VTE Events

**Figure 1:**
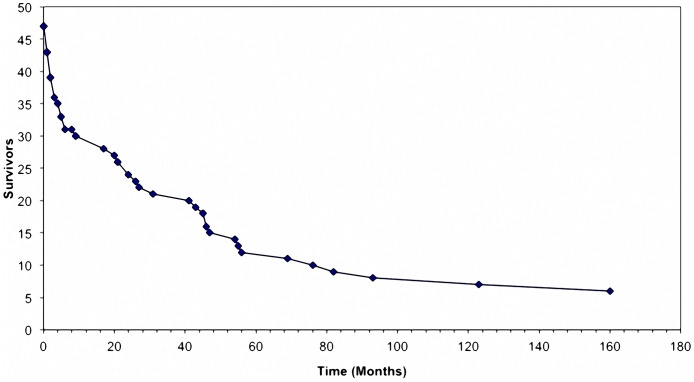
Kaplan Meier curve showing survival of patients who developed VTE with date of VTE as time origin. Thirty-five of 41 (85.3%) patients with VTE died, 20 (57.1%) within 1 year.

Forty-one (9%) patients developed VTE. Of these, 28 (68.3%) had metastatic disease when VTE was diagnosed. Thirty-three patients had deep vein thrombosis (DVT) and 8 had pulmonary embolism, with or without a concomitant diagnosis of DVT. There were no arterial thrombotic events. Twenty-five VTE events (60.9%) were symptomatic and 16 (39.0%) were incidentally diagnosed on imaging. Only 17 (42%) patients were hospitalized at the time of VTE diagnosis. One VTE event (subclavian vein thrombosis) was associated with a central vascular catheter. Thirty five (85.3%) patients with VTE died during the period of follow up. Of these, 20 (57.1%) died within a year of the VTE event (Figure 1). Table 2 summarizes the characteristic of patients with VTE.

**Table 2:** Characteristics of patients with VTE.

Patients who developed VTE	N = 41/453 (9%)
**Stage**	
Limited Stage	3 (7.3)
Locally advanced disease	10 (24.4)
Metastatic disease	28 (68.3)
**Type of presentation**	
DVT	33 (80.5)
Only PE diagnosed	5 (12.2)
Both DVT and PE diagnosed	3 (7.3)
**Symptomatic/Incidental**	
Symptomatic VTE	25 (61.0)*
Incidentally diagnosed VTE	16 (39.0)
**Clinical setting**	
Inpatient	17 (41.5)
Outpatient	24 (58.5)
Central line associated	1 (2.4)
**Location of DVT**	
Subclavian/Axillary	2 (4.9)
Ilio-femoral	19 (46.3)
Popliteal	12 (29.3)
Inferior vena cava	4 (9.8)
Bilateral lower extremity	6 (14.6)

* Five (20%) of 25 symptomatic patients were diagnosed with pulmonary emboli (PE) and 20 (80%) were diagnosed with deep vein thrombosis (DVT) alone while 3 (18.7%) of patients with incidental VTE were diagnosed with PE and 13 (81.3%) with DVT.

We compared the clinical correlates of patients with prostate cancer who developed VTE with those who did not. On univariate analysis, advanced cancer stage, poor performance status (ECOG 3 or 4), chemotherapy, hormonal therapy and the presence of peripheral vascular disease were found to be associated with DVT. On subjecting these factors to multivariate analysis, cancer stage [HR 2.83 (1.43–5.61), p = 0.003) and peripheral vascular disease [HR 4.31 (1.57–11.86), p = 0.005] remained significant predictors for developing VTE. Thromboprophylaxis (for another indication such as atrial fibrillation or mechanical valves) was associated with reduced risk of VTE [HR 0.19 (0.05–0.64), p = 0.008] however patients who were on aspirin were not protected against VTE (HR 1.98 (0.67–3.28), p = 0.078). Other co-morbidities such as congestive heart failure (p = 0.61), chronic obstructive pulmonary disease (p = 0.63), smoking (p = 0.60), obesity (p = 0.20), diabetes mellitus (p = 0.83) and chronic kidney disease (p = 0.14) did not affect VTE risk in this population.

### VTE and Mortality

**Figure 2:**
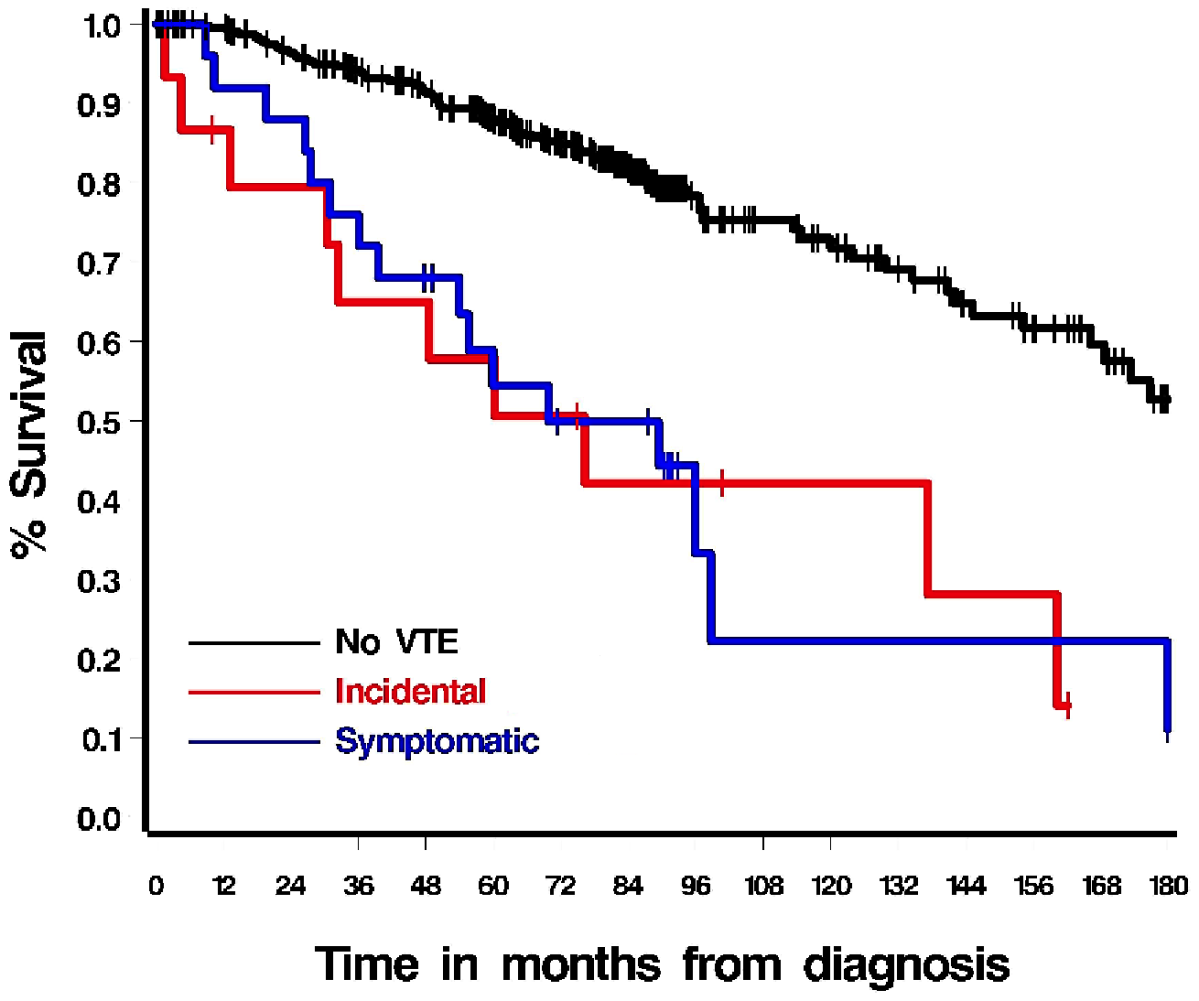
Survival curves depicting survival (in months) for patients with and without venous thromboembolic disease (VTE). Patients with VTE have higher mortality compared to patients without VTE [HR 6.89 (4.29–11.08), p<0.001]. There was no difference in mortality between patients with symptomatic or incidental VTE (p = 0.83).

VTE was found to be significantly associated with mortality [HR 6.89 (4.29–11.08), p<0.001] even when adjusted for other variables such as cancer stage, Gleason grade, performance status, chemotherapy, hormonal therapy, radiation, surgery and other co-morbidities such as congestive heart failure, diabetes mellitus, renal disease, chronic obstructive pulmonary disease, peripheral vascular disease, and other cancers. Figure 2 depicts the survival curves for patients with and without VTE. There was no survival difference between patients with symptomatic or incidental events. The location of thrombosis also did not affect outcomes. On multivariate analysis older age (p<0.001), congestive heart failure (p = 0.002), presence of another malignancy (p = 0.001) and poor performance status (p<0.001) were also associated with decreased survival.

## Discussion

The strong bidirectional link between cancer and VTE has been well described and many studies have described the increased risk of malignancy following VTE, and the subsequent risk of VTE after a diagnosis of cancer. Only recently has attention been focused on survival and morbidity following a thrombotic event in patients with cancer.

In a retrospective cohort study we found a 9% incidence of VTE in patients with prostate cancer over a median follow up of 83 months. Nearly a third of VTE events were diagnosed incidentally. Two thirds of VTE developed in outpatients, but a majority of patients were hospitalized for treatment of VTE potentially leading to increased morbidity and cost.

Metastatic disease and peripheral vascular disease were independent risk factors for developing thrombosis in these patients. While this association has not been previously reported for prostate cancer, metastatic disease has been found to be a risk factor for thrombosis in other cancers [13]. Moreover, recent reports suggest an important association between the risks for development of arterial and venous thrombi based on shared risk factors and pathogenic mechanisms. For example, atherosclerosis is associated with activation of platelets and coagulation and increased fibrin turnover that can lead to venous thrombosis [19]. Some clinical studies have also demonstrated the link between venous thrombosis and peripheral arterial disease. Cogo et al. found that symptomatic peripheral arterial disease was an independent risk factor for idiopathic DVT (odds ratio 1.9, p = 0.04) [20]. Another study reported venous thrombosis in 27/136 (19.9%) patients with symptomatic peripheral arterial disease compared to 2/40 (5%) of controls without PVD [21]. Older age, congestive heart failure, COPD, obesity, diabetes and renal disease have been found to be associated with venous thrombosis in the general population but did not increase risk of VTE in our cohort of patients with prostate cancer. This has also been observed by Prestidge et al. in a group of patients with breast, colon, prostate, lung and pancreatic cancer suggesting that a unique set of risk factors operate in patients with cancer [22].

We found that VTE, whether symptomatic or incidental, had an adverse effect on survival. This has previously been reported in large, heterogeneous cancer populations [4], [5]. In our study, mortality was due to either direct consequences of thrombosis, or more commonly, progressive disease. A similar phenomenon was observed in a trial of 399 patients (79 with cancer) with pulmonary embolism, which reported that progressive malignancy was the most common cause of death in the year after the thromboembolic event [23]. The higher mortality following thromboembolic events cannot be attributed to direct effects of VTE alone and suggests an association between hypercoagulability and progression of the underlying neoplasm.

The pathogenesis of the pro-thrombotic state in cancer is multifactorial. Patient related causes include decreased mobility, the frequent use of vascular catheters and a high incidence of acute medical illnesses and hospitalization. Cancer associated mechanisms include direct vascular invasion and compression by tumor, the effects of chemotherapeutic drugs and increased levels of acute phase reactants such as factor VIII and von Willebrand factor due to cancer related inflammation [13], [24]. The hemostatic system is activated in patients with malignancies, leading not only to hypercoagulability but stimulation of tumor growth and angiogenesis; tissue factor (TF) is a key mediator in this interaction [10]. The up-regulation of TF is a characteristic of malignant cells, and higher TF expression has been associated with increased angiogenesis, metastasis and a poor prognosis in solid cancers such as pancreatic and ovarian cancer [25]–[30]. Studies in prostate cancer have also shown that TF expression was significantly higher in prostate cancer cells than benign prostatic epithelium and correlates significantly with Gleason score and stage of disease, the expression of VEGF-A, higher micro-vessel density, bone metastases and poor survival [11], [31]–[33]. These observations support the hypothesis that thromboembolic disease is more than an epiphenomenon in patients with cancer and that the clinical hypercoagulable state may be a surrogate for an aggressive tumor phenotype.

Clinically unsuspected VTE diagnosed on CT scans is a relatively common problem in the oncologic population [15], [16]. There is no definitive evidence on how these patients should be managed. However, our study demonstrates that in prostate cancer, incidental thrombosis is associated with the same poor outcomes as symptomatic thrombosis, and support the current guidelines of the American College of Chest Physicians that recommend anticoagulation for these patients [34].

This study has some limitations. First, it was retrospective. Patients with apparently asymptomatic VTE may have subtle symptoms such as fatigue or mild shortness of breath prior to the diagnosis. These symptoms may not have been consistently documented unless they were striking. Second, the 9% incidence of thrombotic events in our patient cohort is higher than reported in other series (1.4–2.8%), which may be attributable to the inclusion of patients with incidental VTE which were not included in these other studies, as well as higher proportion of patients with advanced disease referred to a tertiary care center. Thus, our results may not be entirely applicable in all settings. They do however, emphasize that VTE is a significant problem in patients with advanced prostate cancer.

In conclusion, we report that VTE is associated with reduced survival in patients with prostate cancer. Novel findings of this study are the relatively high proportion of incidental VTE (30%) and the association of both symptomatic and incidental VTE with mortality. This may have implications for the management of patients with prostate cancer and demonstrates the need for further studies to evaluate the benefit of prophylactic and therapeutic anticoagulation in this population.
